# Ketamine in the treatment of obsessive-compulsive disorder – a case report and literature review

**DOI:** 10.1192/j.eurpsy.2024.1307

**Published:** 2024-08-27

**Authors:** I. Mangas Palma, I. da Fonseca Pinto, J. Tavares Coelho, R. Moreira

**Affiliations:** Serviço de Psiquiatria, Centro Hospitalar Universitário de São João, Porto, Portugal

## Abstract

**Introduction:**

Obsessive-compulsive disorder (OCD) is a chronic condition characterized by time-consuming and distressing obsessions and/or compulsions, often accompanied by avoidance behaviours. It is a highly prevalent and incident disorder that results in considerable disability and quality of life reduction.

Current pharmacological treatments are hindered by their delayed onset and the limited evidence on how to approach first and second line treatment-resistant patients.

Recent research showcased the involvement of glutamatergic pathways in the pathophysiology of OCD prompting research into the potential therapeutic use of ketamine, which binds to the N-methyl-D-aspartic acid receptor and acts as a non-competitive antagonist of glutamate.

**Objectives:**

The aim of this study is to conduct a literature review on the use of ketamine and its enantiomers as a treatment for OCD and report a clinical case involving an OCD patient who experienced significant improvement following ketamine use.

**Methods:**

A search was performed on PubMed using a combination of keywords and Medical Subject Headings terms, including “Ketamine”, “Esketamine” and “Obsessive-Compulsive Disorder”. Only studies that involved patients with OCD aged ≥18 years who had received ketamine or its enantiomers as an intervention and that reported treatment response using a validated scale were included.

**Results:**

Nine studies were included, 4 case reports, 3 open-label trials and 2 randomized controlled trials, totalling 71 patients. Ketamine was administered intravenously in 7 studies and intranasally in the remaining 2. The results were heterogeneous, with some studies reporting no effect on obsessive-compulsive (OC) symptoms and others demonstrating significant and rapid improvement, albeit some only transitorily.

We present the case of a 42-year-old man who experienced OC symptoms since the age of 20 but was only formally diagnosed with OCD 3 years ago. During his first consultation, the patient described obsessive thoughts related to contamination and dirtiness, accompanied by handwashing rituals and avoidance behaviours (e.g., avoiding touching handles and switches). His Yale-Brown Obsessive Compulsive Scale (Y-BOCS) score was 29. Escitalopram was initiated with a progressive dose titration, resulting in partial improvement (Y-BOCS 23). In a follow-up appointment, the patient disclosed that he had purchased and self-administered a single intravenous dose of 2g of ketamine 2 months earlier for recreational use. This led to an immediate and significant improvement of his OC symptoms. Subsequent re-evaluation 4 months later confirmed that he remained asymptomatic (Y-BOCS 2).

**Conclusions:**

Ketamine may be a therapeutic alternative for OCD patients who are treatment resistant due to its rapid anti-obsessional effect. Further studies with improved designs and larger sample sizes are warranted to better assess the efficacy of ketamine in OCD treatment.

**Disclosure of Interest:**

None Declared

